# Quantification of weekly strength-training volume per muscle group in competitive physique athletes

**DOI:** 10.3389/fspor.2025.1536360

**Published:** 2025-07-30

**Authors:** Thiago Beraldo da Silveira, Gustavo Paula Leite de Almeida, Nelson Carvas Junior, Alexandre Fernandes Machado, Roberta Luksevicius Rica, Francisco Luciano Pontes, Fabiana Rodrigues Scartoni, Valentina Bullo, Stefano Gobbo, Marco Bergamin, Danilo S. Bocalini, Aylton Figueira Júnior, Gustavo Allegretti João

**Affiliations:** ^1^ Department of Biological Sciences and Health, São Judas Tadeu University, São Paulo, Brazil; ^2^ Institute of Health Sciences, Paulista University, São Paulo, Brazil; ^3^ Center of Physical Education and Sport, Federal University of Espiríto Santo, Vitória, Brazil; ^4^ Physiology of Exercise & Aging Laboratory, São Paulo University, São Paulo, Brazil; ^5^ Sport and Exercise Science Laboratory, University Catolic of Petrópolis, Rio de Janeiro, Brazil; ^6^ Department of Medicine, University of Padua, Padova, Italy; ^7^ Department of Physical Education, United Metropolitan Colleges, São Paulo, Brazil

**Keywords:** competitive bodybuilders, bodybuilding training, physique athletes training, strength training, volume training, resistance training

## Abstract

The purpose of the study was to quantify and compare the volume of training performed by competitive physique athletes, obtaining unprecedented findings at this level of specificity given the scarce specific literature and comparing with guidelines, in order to generate more specific and accurate conducts in the future.

**Methods:**

One hundred and fifty-four athletes from different federations and categories were analyzed using questions with items about training strategies describing them and subsequently comparing them with each other and with guidelines.

**Results:**

Many of the categories reduced training volume in pré-contest for most muscle groups. There is a variation in the average number of workouts per week between the off-season and the pré-contest period in different muscle groups. There was a significant difference in the weekly sets volume in categories (*p* < 0.05, *α* = 0,05): Men's Physique (MP) reduced (pectoral); Classic Physique (CP) and MP reduced (deltoids); Bodybuilding Classic (BC) vs. MP (pectoral) off-season and BC vs. MP (deltoids) off-season; Master (MT) increased (quadriceps); BC and MT increased (abdominals); CP vs. MP pré-contest (triceps surae). There was a significant increase in the duration of cardio work in pré-contest: CP, BC, Bikini (BK), and Wellness (WL) (*p* < 0.05, *α* = 0,05). An increase was observed as statistically significant in the weekly frequency of BC and Senior (SN) (*p* < 0.05). In most cases, there was an increase in the amount of cardio work during the pré-contest period.

**Conclusion:**

Notable reductions in training volume were observed during the pré-contest period for some muscle groups. Both men and women exhibited a decrease in exercise frequency for some muscle groups during pré-contest, alongside an increase in cardio training. We suggest new studies that can help with developing more detailed training practices for physique athletes.

## Introduction

Physique Athletes maintain lean mass while reducing body fat levels during competition preparation with high resistance training and increased aerobic training (1) Strength training (ST) is a well-established intervention strategy for increasing muscle Mass. A hypertrophy-oriented program should employ a repetition range of 6–12 reps per set with rest intervals of 60­90 s between sets. Exercises should be varied in a multiplanar, multiangled fashion to ensure maximal stimulation of all muscle fibers. Multiple sets should be employed in the context of a split training routine to heighten the anabolic milieu (2).

In off-season most physique athletes use split routines (85.5%), train 4–7 times per week, target major muscle groups twice weekly, and sessions last 60–90 min. Typical sessions involve 2–3 muscle groups, 2–3 exercises per group, 3–4 sets per exercise, 7–12 repetitions per set, and 61–180 s of rest between sets. Six weeks before competition, there is a shift to fewer muscle groups per session, more repetitions per set, and increased aerobic exercise. Rest periods become shorter (30–60 s), and training intensity and volume are adjusted to enhance muscle definition, though this may risk muscle mass loss, especially in natural physique athletes (3, 4). It is widely recognized that manipulating ST variables is crucial for maximizing hypertrophic adaptations.

The single workout must then be designed reflecting these targeted program goals including the choice of exercises, order of exercise, amount of rest used between sets and exercises, number of repetitions and sets used for each exercise, and the intensity of each exercise (5).

Among these variables, training volume—commonly defined as the number of sets performed per muscle group per week—is considered one of the most critical factors. Consequently, numerous systematic reviews (6), meta-analyses (7, 8), and position statements (9, 10) have sought to establish evidence-based guidelines for the optimal number of sets to promote muscle hypertrophy across diverse populations (11) recommended that natural physique athletes perform approximately 40–70 repetitions per session at least twice per week, noting that higher volumes might be suitable for more advanced lifters.

A meta-analysis by (8) found that performing at least 10 sets per muscle group per week resulted in greater increases in muscle mass compared to fewer than 10 sets, but only two studies included in the meta-analysis had investigated the effects of RT volume on changes in muscle mass specifically in resistance-trained individuals.

However, the analysis lacked sufficient data to determine whether additional hypertrophicbenefits could be achieved with volumes exceeding this threshold. More recently (12), suggested limiting weekly volume to fewer than 15 sets per muscle group, proposing that higher volumes may impair recovery and thereby diminish muscular adaptations. The American College of Sports Medicine recommends three sets of 8–12 repetitions per major muscle group for general fitness in healthy adults (9). Similarly, the National Strength and Conditioning Association suggests 1–3 sets of 6–15repetitions for strength training in youth populations (13).

Despite these efforts to provide evidence-based recommendations, no studies to date havespecifically examined whether these guidelines align with the training practices ofphysique athletes, whose primary objective is to maximize muscle mass (8). We consider this increase casually according to the needs of each athlete.

Hypothetically believe that many practices are in accordance with the literature but can be more detailed and refined according to the specific needs of each category and training period, sincethere are research in this direction is relevant to the theory and practice of bodybuilding, indicating an important gap in the literature on how to adapt training programs to the individual characteristics of athletes (14). Many practices recommended by coaches still lack robust scientific validation, highlighting the need for more studies to support such recommendations (15).

Knowledge of practices in more specific ways becomes essential, because while criteria exist, their application can be influenced by subjective interpretation—both by athletes and judges. The way a body is “read” or evaluated often depends on how well it aligns with these standards, but also on the embodied knowledge and presentation of the athlete, The body's response to training is both a physical and subjective process.

Athletes adjust their routines based on how their bodies feel and react, which in turn shapes how they meet the criteria (16). Therefore this study aimed to: (1) quantify the weekly training volume performed by competitive physique athletes, (2) identify potential differences between male and female athletes, (3) compare training practices acrosscompetitive categories for different muscle groups, and (4) and to critically assess whether current recommendations are sufficient to inform competitive athlete practice.

## Materials and methods

### Study design

An online, cross-sectional survey study was conducted between April and December 2020. Data collection utilized an online questionnaire administered via Google Forms, available in two languages (English and Portuguese). The survey was distributed throughsocial media platforms and the professional and personal networks of the authors. There are no guidelines or consensus on the weekly volume specifically for different categories per period, so the objective is to analyze training practices by collecting data from the off-season and pré-contest periods.

### Participants and population

A total of one hundred and fifty-four competitive physique athletes consented to participate in the study, allowing the analysis of their current strength training (ST) programs during both off-season and pré-contest periods.

All participants provided informed consent, allowing the analysis of their ST programs. The study adhered to ethical standards for research involving human subjects, following the principles outlined in the Declaration of Helsinki. The project received approval from the Ethics Committee of the University São Judas Tadeu (CAAE: 66523917.1.0000.0089; approval report number: 2.022.898).

Participants were eligible if they met the following criteria:

Being affiliated with a federation or organization, having participated in at least one competition in the last two years, being male or female, in addition to maintaining a training routine throughout the collection period, have their practices written down or spreadsheeted.
Exclusion criteria were used for athletes who did not read or answer all the questions, and athletes who did not participate in the competition.

Participants were affiliated with a range of competitive organizations, including:

• International Federation of Body Building and Fitness – São Paulo (IFBB SP): 1 athlete
• São Paulo Fisiculturismo e Fitness (SPFF): 19 athletes
• National Physique Committee PRO LEAGUE (NPC): 85 athletes
• International Federation of BodyBuilding and Fitness ELITE PRO (IFBB ELITE PRO): 39 athletes
• National Amateur Bodybuilders’ Association (NABBA): 1 athlete
• World Beauty Fitness & Fashion (WBFF): 2 athletes
• World Beauty Fitness & Fashion Professional (WBFF PRO): 1 athlete
• World Fitness Federation (WFF): 1 athlete
• Other affiliations: 5 athletes

### Data collection

To ensure transparency, a dummy version of the survey is publicly accessible for readers to review the specific questions presented to respondents (https://linktr.ee/pesquisausjt).

Table 1 presents a comparison of body mass across all participants competitive categories, alongside the anthropometric characteristics and the number of subjects evaluated in each category. Significant variations in body mass were observed between the off-season and pré-contest phases within each category.

**Table 1:** Frequencies for categories, and characteristics anthropometric (mean ± SD).

*n* = 154	Categories	Off-season	Pré-contest	Height
11	Master	101.56 ± 10.86	89.18 ± 11.83	176.73 ± 7.27
14	Bodybuilding Classic	93.00 ± 15.75	76.43 ± 8.87	172.86 ± 5.19
26	Classic Physique	95.90 ± 11.42	79.36 ± 10.92	174.33 ± 6.06
18	Senior	103.27 ± 12.94	88.76 ± 10.82	172.67 ± 5.49
50	Men's Physique	88.60 ± 9.97	78.25 ± 10.37	173.64 ± 7.02
20	Wellness	66.07 ± 7.10	59.95 ± 5.38	162.45 ± 4.87
3	Women's Physique	80.50 ± 6.36	63.33 ± 4.51	158.67 ± 9.02
9	Bikini	62.00 ± 4.00	52.56 ± 3.40	167.11 ± 7.80
3	Figure	60.00 ± 7.07	57.00 ± 6.24	160.67 ± 8.14
		Body mass		

### Data analysis

#### Weekly training volume by muscle group

The weekly training volume for each muscle group was calculated based on equations used in prior studies (17, 18).

Numberofexercisespermusclegrouppertrainingsession×Numberofsetsperexerciseineachtrainingsession×Weeklytrainingfrequencypermusclegroup

The study focused on analyzing the most commonly trained muscle groups in hypertrophy-oriented programs: pectoralis major, latissimus dorsi, deltoids, biceps brachii, triceps brachii, gluteal muscles, quadriceps, hamstrings, gastrocnemius (triceps surae), and abdominal muscles. For each muscle group, commonly performed exercises (e.g., French press for the triceps brachii) were included in the analysis. Table 2 provides a comprehensive list of exercises considered for each muscle group. All variations of these exercises were included, regardless of the training modality utilized, such as free weights, machines, pulley systems, or bodyweight exercises.

**Table 2:** Exercises cited and included in the analysis per muscle group.

Muscle group	Exercises analysis (free weight or machine)
Pectoral	Bench press (all variations with wide grip)
	Fly (all variations)
	Crossover/pec deck
	Pullover
	Pulldown (all variations)
	Pull up
Latissimus dorsi	Lat pull down
	Rowing (all variations with closed grip)
	Graviton
	Deadlift
	Shoulder press (all variations)
Deltoid	Upright row
	Raise (lateral, frontal)
Biceps brachii and Triceps brachii	Elbow curl (all variations)
	Elbow extension (all variations)
	Hip extension (all variations)
	Squat (all variations)
Gluteal/Quadriceps	Leg press
	Lunge (all variations)
	Hip thrust
	Knee extension (all variations)
	Knee curl (all variations)
Hamstrings	Stiff deadlift
	Nordic hamstrings
	Trunk flexion (all variations)
Triceps suraes	Ankle extension (all variations)

### Statistical analysis

Descriptive statistics were presented as mean ± standard deviation (SD), median (Med), minimum value (Min), and maximum value (Max). The Shapiro–Wilk test indicated that the data were not normally distributed. To assess differences within groups (categories), the Wilcoxon test, Kruskal–Wallis test, and *post-hoc* Kruskal–Nemenyi test were employed.

Non-parametric tests were employed to assess differences between categories or within groups. The Wilcoxon test was used for comparisons between two groups or conditions. For comparisons among three or more independent groups, the Kruskal–Wallis test was applied. When the Kruskal–Wallis test indicated statistical significance, *post-hoc* Kruskal–Nemenyi tests were conducted for pairwise comparisons.

For all statistically significant tests, effect sizes were calculated to quantify the magnitude of observed differences. For the Kruskal–Wallis test, effect size was reported as eta-squared (*η*_H_^2^). For the Wilcoxon test, appropriate rank-based effect size measures were considered to provide insight into the practical relevance of the findings.

Power calculations for the chi-squared test were performed using R (version 3.6.0; 2019) and RStudio (version 1.2.1335; 2019) via the power.chisq command. An *a priori* power analysis, assuming an error rate of *α* = 0.05, 80% power, and 4 degrees of freedom (*df*), yielded an effect size of 0.80. Based on these parameters, a minimum sample size of 19 participants was estimated to detect a statistically significant difference.

Statistical significance was set at *p* ≤ 0.05. All analyses were conducted using R (version 3.6.0; 2019) and RStudio (version 1.2.1335; 2019).

## Results

Table 3 compares the frequency of exercises performed for different muscle groups during the off-season and pré-contest phases.

**Table 3:** Frequencies of exercises during off-season and pré-contest.

Exercises	Men		Women	
	Off-season	Pré-contest	Off-season	Pré-contest
	(%)	(%)	(%)	(%)
1. Bench press	62	58	37	34
2. Pec Deck	55	54	17	20
3. Incline Bench press	54	55	3	11
4. Incline fly	53	45	11	17
5. Lat pull down	78	77	49	51
6. Barbell row lats	73	66	40	46
7. Seated row cable	56	52	23	31
8. One-arm dumbbell lat row	55	58	43	49
9. Pull up	48	39	17	11
10. Dumbbells lateral raises	81	76	60	57
11. Dumbbells shoulder press	60	53	49	60
12. Dumbbells front raises	59	52	46	46
13. Machine shoulder press	43	49	29	34
14. Rear deltoid dumbbells or machine	45	43	14	23
15. Shrug exercise dumbbells/machine	39	37	3	14
16. Barbell curl	49	43	40	51
17. Barbell “W” curl	48	47	17	17
18. Dumbbell concentration curl	43	33	31	23
19. Scott machine	38	32	17	20
20. Cable triceps extension	75	75	37	49
21. Barbell “W” triceps extension	49	39	17	20
22. Leg Press 45°	77	73	66	66
23. Squat	73	69	66	74
24. Leg extension machine	72	76	63	71
25. Hamstring leg curl machine	83	81	71	77
26. Seated hamstring leg curl machine	61	74	63	69
27. Stand leg curl alternative hamstring	52	55	49	77
28. Stiff Deadlift	60	55	71	77
29. Hip thrust	45	38	71	80
30. Gluteus machine	19	17	49	49
31. Hip abduction machine	60	55	63	77
32. Hip adduction machine	42	38	49	46
33. Seated calf	82	73	57	69
34. Abs raise leg	45	44	37	43
35. Back extension	65	59	46	51
	Men		Women	
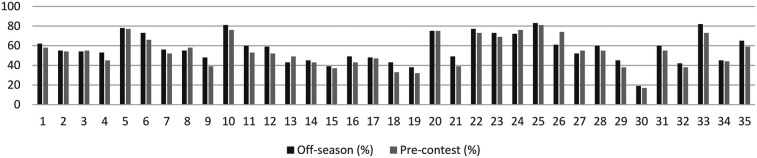				
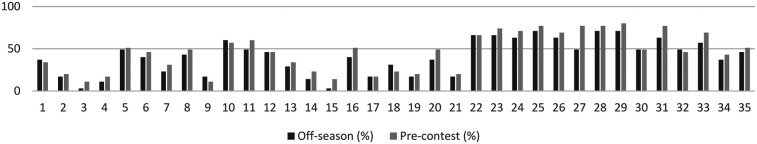				

During the off-season, the most frequently performed exercises among men were lat pulldowns (78%), 45° leg press (77%), squats (73%), seated hamstring curls (83%), and seated calf raises (82%). During the pré-contest phase, the most common exercises were squats (69%), seated hamstring curls (81%), 45° leg press (73%), seated calf raises (73%), and back extensions (59%) (Table 3).For women, the most frequent off-season exercises were seated hamstring curls (71%), squats (66%), leg extensions (63%), lat pulldowns (49%), and hip abduction machine exercises (63%). During the pré-contest phase, the most frequently performed exercises were squats (74%), seated hamstring curls (77%), leg extensions (71%), hip thrusts (80%), and hip adduction machine exercises (46%). When comparing men and women during the off-season, men were found to perform certain exercises more frequently, including bench press (62% of men vs. 37% of women), pec deck (55% vs. 17%), incline bench press (54% vs. 3%), and barbell rows (73% vs. 40%).

Similar trends were observed during the pré-contest phase, where men demonstrated a higher frequency of bench press (58% of men vs. 34% of women), pec deck (54% vs. 20%), incline bench press (55% vs. 11%), and barbell rows (66% vs. 46%) compared to women.

Tables 4, 5 present the statistical analyses by category, comparing the weekly set volume performed for the upper body during the off-season and pré-contest phases in both men's and women's categories.

**Table 4:** Comparison (*P*-value) of weekly sets volume performed to upper body per week in off-season and pré-contest in categories men.

Period	Categories	Kruskal's Test		*Δ*%	Wilcoxon's Test	Kruskal's Test		Post hoc	
Group muscle		Off-season	Pré-contest			Off-season	Pré-contest	Off-season	Pré-contest
Pectoral	Classic Physique	27.00 (4.00–80.00)	29.00 (4.00–80.00)	7.41	*p*-value = 0.92	*p*-value = 0.00 *η*^2^ 0.09 moderate	*p*-value = 0.18 *η*_H_^2^ 0.02 small		
	Bodybuilding Classic	20.00 (5.00–33.00)	25.00 (5.00–40.00)	25	*p*-value = 0.28			d	
	Master	25.00 (8.00–70.00)	24.00 (14.00–30.00)	−4	*p*-value = 0.85				
	Men's Physique	32.00 (8.00–70.00)	31.00 (12.00–56.00)	−3.13	*p*-value = 0.00*			b	
	Senior	22.00 (4.00–48.00)	24.00 (5.00–50.00)	9.09	*p*-value = 0.21				
Latissimus dorsi	Classic Physique	29.00 (12.00–65.00)	30.00 (12.00–65.00)	3.45	*p*-value = 0.73	*p*-value = 0.12 *η*_H_^2^ 0.03 small	*p*-value = 0.59 *η*_H_^2^−0.01 small		
	Bodybuilding Classic	24.00 (4.00–40.00)	30.00 (5.00–60.00)	25	*p*-value = 0.13				
	Master	30.00 (8.00–80.00)	24.00 (14.00–60.00)	−20	*p*-value = 0.71				
	Men's Physique	32.00 (8.00–70.00)	32.00 (12.00–56.00)	0	*p*-value = 0.03				
	Senior	24.00 (5.00–48.00)	28.00 (5.00–50.00)	16.67	*p*-value = 0.10				
Deltoid	Classic Physique	26.00 (6.00–62.00)	24.50 (8.00–48.00)	−5.77	*p*-value = 0.02*	*p*-value = 0.02 *η*_H_^2^−0.07 moderado	*p*-value = 0.40 *η*_H_^2^ 0.00 small		
	Bodybuilding Classic	22.00 (2.00–32.00)	24.00 (4.00–48.00)	9.09	*p*-value = 0.27			d	
	Master	18.00 (6.00–50.00)	20.00 (12.00–40.00)	11.11	*p*-value = 0.28				
	Men's Physique	33.50 (6.00–80.00)	30.00 (0.00–48.00)	−10.45	*p*-value = 0.00*			b	
	Senior	20.00 (5.00–45.00)	24.00 (6.00–50.00)	20	*p*-value = 0.31				
Biceps Brachii	Classic Physique	16.00 (3.00–36.00)	16.00 (4.00–36.00)	0	*p*-value = 0.75	*p*-value = 0.17 *η*_H_^2^ 0.02 small	*p*-value = 0.49 *η*_H_^2^−0.00 small		
	Bodybuilding Classic	12.00 (2.00–24.00)	14.00 (4.00–32.00)	16.67	*p*-value = 0.89				
	Master	15.00 (4.00–24.00)	16.00 (10.00–24.00)	6.67	*p*-value = 0.28				
	Men's Physique	18.00 (0.00–32.00)	14.00 (0.00–32.00)	−22.22	*p*-value = 0.09				
	Senior	19.00 (3.00–30.00)	20.00 (3.00–48.00)	5.26	*p*-value = 0.22				
Triceps Brachii	Classic Physique	16.00 (4.00–36.00)	16.00 (4.00–36.00)	0	*p*-value = 0.89	*p*-value = 0.42 *η*_H_^2^−0.00 small	*p*-value = 0.16 *η*_H_^2^ 2 0.02 small		
	Bodybuilding Classic	12.00 (2.00–24.00)	12.00 (4.00–32.00)	0	*p*-value = 0.85				
	Master	16.00 (5.00–30.00)	18.00 (12.00–40.00)	12.5	*p*-value = 0.06				
	Men's Physique	16.00 (4.00–40.00)	16.00 (4.00–32.00)	0	*p*-value = 0.29				
	Senior	18.00 (3.00–30.00)	20.00 (3.00–48.00)	11.11	*p*-value = 0.17				

Mean (minimal–maximum); statistic difference by posthoc.kruskal.nemenyi.test between: a, class; b, bodybuilding classic; c, master; d, men's physique; e, senior.

* *p* < 0.05.

**Table 5:** Comparison (*P*-value) of weekly sets volume performed to upper body per week in off-season and pré-contest in categories women.

Group muscle	Categories	Period		*Δ*%	Wilcoxon's Test	Kruskal's Test		Post hoc	
		Off-season	Pré-context			Off-season	Pré-contest	Off-season	Pré-contest
Pectoral	Bikini	7.50 (0.00–24.00)	8.00 (0.00–12.00)	6.67	*p*-value = 0.32	*p*-value = 0.28 *η*_H_^2^ 0.03 small	*p*-value = 0.14 *η*_H_^2^ 0.08 moderate		
	Figure	12.00 (8.00–16.00)	16.00 (8.00–26.00)	33.33	*p*-value = NA				
	Wellness	1.00 (0.00–16.00)	1.50 (0.00–16.00)	50	*p*-value = 0.20				
	Women's Physique	2.00 (0.00–4.00)	4.00 (0.00–20.00)	100	*p*-value = NA				
Latissimus dorsi	Bikini	12.00 (1.00–25.00)	12.00 (1.00–44.00)	0	*p*-value = 0.65	*p*-value = 0.91 *η*_H_^2^−0.08 moderate	*p*-value = 0.64 *η*_H_^2^−0.04 small		
	Figure	12.00 (0.00–24.00)	24.00 (0.00–30.00)	100	*p*-value = NA				
	Wellness	16.00 (0.00–32.00)	16.00 (0.00–36.00)	0	*p*-value = 0.68				
	Women's Physique	17.00 (4.00–30.00)	30.00 (4.00–48.00)	76.47	*p*-value = NA				
Deltoid	Bikini	7.50 (2.00–48.00)	15.00 (2.00–48.00)	100	*p*-value = 0.18	*p*-value = 0.98 *η*_H_^2^−0.09 moderate	*p*-value = 0.63 *η*_H_^2^−0.04 small		
	Figure	23.00 (16.00–30.00)	30.00 (16.00–32.00)	30.43	*p*-value = NA				
	Wellness	18.00 (0.00–50.00)	17.00 (0.00–50.00)	−5.56	*p*-value = 0.31				
	Women's Physique	20.00 (4.00–36.00)	36.00 (4.00–40.00)	80	*p*-value = NA				
Biceps Brachii	Bikini	7.50 (0.00–18.00)	8.00 (0.00–24.00)	6.67	*p*-value = 1	*p*-value = 0.59 *η*_H_^2^−0.03 small	*p*-value = 0.20 *η*_H_^2^ 0.05 small		
	Figure	12.50 (12.00–13.00)	13.00 (12.00–16.00)	4	*p*-value = NA				
	Wellness	8.00 (0.00–24.00)	7.00 (0.00–24.00)	−12.05	*p*-value = 0.32				
	Women's Physique	11.00 (4.00–18.00)	18.00 (4.00–36.00)	63.64	*p*-value = NA				
Triceps Brachii	Bikini	7.50 (0.00–18.00)	9.00 (0.00–16.00)	20	*p*-value = 0.18	*p*-value = 0.92 *η*_H_^2^−0.08 moderate	*p*-value = 0.22 *η*_H_^2^ 0.45 small		
	Figure	12.50 (12.00–12.00)	12.00 (12.00–20.00)	−4	*p*-value = NA				
	Wellness	10.00 (0.00–26.00)	8.50 (0.00–26.00)	−15	*p*-value = 0.23				
	Women's Physique	11.00 (4.00–18.00)	18.00 (4.00–36.00)	63.64	*p*-value = NA				

Mean (minimal–maximum); statistic difference by posthoc.kruskal.nemenyi.test between: a, wellness; b, women's physique; c, bikini; d, figure.

Tables 6, 7 compare the weekly set volume performed for the lower body and core muscles during the off-season and pré-contest phases across men's and women's categories.

**Table 6:** Comparison (*P*-value) of weekly sets volume performed to lower body and CORE per week in off-season and pré-contest in categories men.

Group muscle	Categories	Period		*Δ*%	Wilcoxon's Test	Kruskal's Test		Post hoc	
		Off-season	Pré-context			Off-season	Pré-contest	Off-season	Pré-contest
Quadriceps	Classic Physique	24.00 (8.00–42.00)	28.00 (18.00–42.00)	16.67	*p*-value = 0.61	*p*-value = 0.22 *η*_H_^2^ 0.01 small	*p*-value = 0.07 *η*_H_^2^ 0.04 small		
	Bodybuilding Classic	16.00 (4.00–40.00)	20.00 (4.00–48.00)	25	*p*-value = 0.40				
	Master	20.00 (7.00–80.00)	24.00 (14.00–40.00)	20	*p*-value = 0.04*				
	Men's Physique	22.00 (1.00–56.00)	22.00 (10.00–36.00)	0	*p*-value = 0.50				
	Senior	22.50 (5.00–60.00)	20.00 (6.00–60.00)	−11.11	*p*-value = 0.63				
Hamstrings	Classic Physique	19.00 (8.00–36.00)	20.00 (12.00–36.00)	5.26	*p*-value = 0.83	*p*-value = 0.67 *η*_H_^2^−0.01 small	*p*-value = 0.55 *η*_H_^2^−0.00 small		
	Bodybuilding Classic	14.00 (2.00–32.00)	16.00 (3.00–40.00)	14.29	*p*-value = 0.92				
	Master	16.00 (6.00–60.00)	16.00 (15.00–40.00)	0	*p*-value = 0.11				
	Men's Physique	19.00 (8.00–50.00)	16.00 (6.00–36.00)	−15.79	*p*-value = 0.91				
	Senior	18.00 (4.00–60.00)	20.00 (5.00–48.00)	11.11	*p*-value = 0.67				
Gluteal	Classic Physique	12.00 (0.00–45.00)	12.00 (0.00–45.00)	0	*p*-value = 0.46	*p*-value = 0.53 *η*_H_^2^−0.00 small	*p*-value = 0.34 *η*_H_^2^ 0.00 small		
	Bodybuilding Classic	7.00 (0.00–30.00)	8.00 (0.00–50.00)	14.29	*p*-value = 0.07				
	Master	10.00 (0.00–20.00)	12.00 (0.00–20.00)	20	*p*-value = 0.11				
	Men's Physique	9.00 (0.00–30.00)	10.00 (0.00–18.00)	11.11	*p*-value = 0.75				
	Senior	7.00 (0.00–24.00)	4.50 (0.00–60.00)	−35.71	*p*-value = 0.41				
Triceps surae	Classic Physique	20.00 (0.00–70.00)	24.00 (0.00–80.00)	20	*p*-value = 0.07	*p*-value = 0.19 *η*_H_^2^ 0.01 small	*p*-value = 0.02 *η*_H_^2^ 0.07 moderate		d
	Bodybuilding Classic	14.00 (1.00–32.00)	16.00 (1.00–50.00)	14.29	*p*-value = 0.34				
	Master	18.00 (4.00–100.00)	18.00 (15.00–100.00)	0	*p*-value = 0.18				
	Men's Physique	16.00 (4.00–50.00)	16.00 (4.00–70.00)	0	*p*-value = 0.19				a
	Senior	16.00 (3.00–50.00)	16.00 (0.00–48.00)	0	*p*-value = 0.12				
Abdominals	Classic Physique	9.00 (0.00–50.00)	14.00 (0.00–50.00)	55.56	*p*-value = 0.15	*p*-value = 0.07 *η*_H_^2^ 0.04 small	*p*-value = 0.54 *η*_H_^2^−0.00 small		
	Bodybuilding Classic	4.50 (0.00–30.00)	12.00 (0.00–70.00)	166.67	*p*-value = 0.01*				
	Master	10.00 (2.00–27.00)	20.00 (8.00–40.00)	100	*p*-value = 0.03*				
	Men's Physique	15.00 (0.00–50.00)	14.00 (0.00–48.00)	−6.67	*p*-value = 0.63				
	Senior	11.00 (0.00–48.00)	12.00 (0.00–80.00)	9.09	*p*-value = 0.44				
Lower back	Classic Physique	3.50 (0.00–12.00)	4.50 (0.00–24.00)	28.57	*p*-value = 0.06	*p*-value = 0.26 *η*_H_^2^ 0.01 small	*p*-value = 0.59 *η*_H_^2^−0.01 small		
	Bodybuilding Classic	4.00 (0.00–30.00)	4.00 (0.00–30.00)	0	*p*-value = 0.44				
	Master	5.00 (2.00–15.00)	8.00 (3.00–20.00)	60	*p*-value = 0.04				
	Men's Physique	4.00 (0.00–40.00)	5.00 (0.00–16.00)	25	*p*-value = 0.73				
	Senior	4.00 (0.00–10.00)	6.00 (0.00–20.00)	50	*p*-value = 0.28				

Mean (minimal–maximum); statistic difference by posthoc.kruskal.nemenyi.test between: a, classic physique; b, bodybuilding classic; c, master; d, men's physique; e, senior.

* *p* < 0.05.

**Table 7:** Comparison (*P*-value) of weekly sets volume performed to lower body and CORE per week in off-season and pré-contest in categories women.

Group muscle	Categories	Period		*Δ*%	Wilcoxon's Test	Kruskal's Test		Post hoc	
		Off-season	Pré-context			Off-season	Pré-contest	Off-season	Pré-contest
Quadriceps	Bikini	7.50 (1.00–20.00)	7.00 (0.00–50.00)	−6.67	*p*-value = 0.18	*p*-value = 0.13 *η*_H_^2^ 0.08 moderate	*p*-value = 0.06 *η*_H_^2^ 0.13 moderate		
	Figure	28.50 (23.00–34.00)	34.00 (23.00–45.00)	19.30	*p*-value = NA				
	Wellness	28.00 (1.00–60.00)	28.00 (2.00–50.00)	0	*p*-value = 0.75				
	Women's Physique	17.00 (4.00–30.00)	30.00 (8.00–36.00)	76.47	*p*-value = 0.32				
Hamstrings	Bikini	17.00 (2.00–32.00)	16.00 (2.00–32.00)	−5.88	*p*-value = 0.32	*p*-value = 0.42 *η*_H_^2^−0.00 small	*p*-value = 0.17 *η*_H_^2^ 0.06 moderate		
	Figure	27.00 (14.00–40.00)	40.00 (14.00–45.00)	48.15	*p*-value = NA				
	Wellness	24.00 (1.00–40.00)	25.50 (1.00–40.00)	6.25	*p*-value = 0.41				
	Women's Physique	11.00 (4.00–18.00)	24.00 (8.00–36.00)	118.18	*p*-value = 0.18				
Gluteal	Bikini	31.00 (2.00–41.00)	15.00 (2.00–32.00)	−51.61	*p*-value = 0.65	*p*-value = 0.34 *η*_H_^2^ 0.01 small	*p*-value = 0.19 *η*_H_^2^ 0.05 small		
	Figure	13.50 (13.00–14.00)	14.00 (13.00–15.00)	3.70	*p*-value = NA				
	Wellness	24.00 (3.00–90.00)	24.00 (1.00–90.00)	0	*p*-value = 0.71				
	Women's Physique	11.00 (4.00–18.00)	18.00 (4.00–24.00)	63.64	*p*-value = 0.31				
Triceps surae	Bikini	8.00 (3.00–24.00)	16.00 (1.00–30.00)	100	*p*-value = 0.28	*p*-value = 0.68 *η*_H_^2^−0.05 small	*p*-value = 0.93 *η*_H_^2^−0.08 moderate		
	Figure	17.50 (17.00–18.00)	17.00 (8.00–18.00)	−2.86	*p*-value = NA				
	Wellness	12.00 (0.00–36.00)	13.00 (0.00–36.00)	8.33	*p*-value = 0.31				
	Women's Physique	9.50 (5.00–14.00)	14.00 (4.00–40.00)	47.37	*p*-value = 0.32				
Abdominals	Bikini	4.00 (0.00–6.00)	5.00 (0.00–12.00)	25	*p*-value = 0.32	*p*-value = 0.12 *η*_H_^2^ 0.09 moderate	*p*-value = 0.23 *η*_H_^2^ 0.04 small		
	Figure	12.00 (0.00–24.00)	12.00 (0.00–24.00)	0	*p*-value = NA				
	Wellness	12.00 (0.00–44.00)	13.50 (0.00–44.00)	12.5	*p*-value = 0.78				
	Women's Physique	2.00 (0.00–4.00)	8.00 (5.00–20.00)	300	*p*-value = 0.18				
Lower back	Bikini	2.50 (0.00–5.00)	4.00 (0.00–12.00)	60	*p*-value = 0.11	*p*-value = 0.89 *η*_H_^2^−0.07 moderate	*p*-value = 0.66 *η*_H_^2^−0.04 small		
	Figure	1.50 (0.00–3.00)	3.00 (0.00–4.00)	100	*p*-value = NA				
	Wellness	1.00 (0.00–18.00)	1.00 (0.00–18.00)	0	*p*-value = 0.28				
	Women's Physique	2.00 (0.00–4.00)	4.00 (0.00–20.00)	100	*p*-value = NA				

Mean (minimal–maximum); statistic difference by posthoc.kruskal.nemenyi.test between: a, wellness; b, women's physique; c, bikini; d, figure.

Table 8 shows that athletes in the “Bikini” category performed cardio exercises at a higher average weekly frequency compared to athletes in the “Women's Physique” category during both the off-season and pré-contest phases. Additionally, during the pré-contest phase, athletes in the “Classic Physique” category performed cardio exercises at a higher average weekly frequency compared to athletes in the “Senior” category.

**Table 8:** Cardio work training off-season and pré-contest.

Categories	Weekly frequency		*Δ*%	Wilcoxon's Test	Kruskal's Test		Post hoc	
	Off-season	Pré-contest			Off-season	Pré-contest	Off-season	Pré-contest
Classic Physique	5.00 (0.00–7.00)	6.50 (0.00–7.00)	30	*p*-value = 0.10	*p*-value = 0.15 *η*_H_^2^ 0.02 small	*p*-value = 0.90 *η*_H_^2^−0.02 small		
Bodybuilding Classic	3.00 (0.00–7.00)	7.00 (0.00–7.00)	133.33	*p*-value = 0.04*				
Master	3.00 (0.00–7.00)	5.00 (0.00–10.00)	66.67	*p*-value = 0.22				
Men's Physique	5.00 (0.00–14.00)	6.00 (0.00–14.00)	20	*p*-value = 0.00				
Senior	3.00 (0.00–7.00)	5.00 (0.00–14.00)	66.67	*p*-value = 0.01*				
Bikini	4.00 (0.00–14.00)	7.00 (5.00–14.00)	75	*p*-value = 0.07	*p*-value = 0.34 *η*_H_^2^ 0.01 small	*p*-value = 0.01 *η*_H_^2^ 0.24 large		d
Figure	3.50 (0.00–7.00)	3.00 (0.00–7.00)	−14.29	*p*-value = NA				
Wellness	6.00 (0.00–7.00)	6.50 (0.00–7.00)	8.33	*p*-value = 0.25				
Women's Physique	1.50 (0.00–3.00)	1.50 (0.00–3.00)	0	*p*-value = NA				a
Categories	During the session–min			Wilcoxon's Test	Kruskal's Test		Post hoc	
	Off-season	Pré-contest			Off-season	Pré-contest	Off-season	Pré-contest
Classic Physique	40.00 (0.00–60.00)	45.00 (0.00–110.00)	12.50	*p*-value = 0.01*	*p*-value = 0.57 *η*_H_^2^ -−0.00 small	*p*-value = 0.78 *η*_H_^2^−0.02 small		
Bodybuilding Classic	30.00 (0.00–60.00)	40.00 (0.00–60.00)	33.33	*p*-value = 0.03*				
Master	30.00 (0.00–60.00)	45.00 (0.00–60.00)	50	*p*-value = 0.28				
Men's Physique	30.00 (0.00–75.00)	40.00 (0.00–75.00)	33.33	*p*-value = 0.15				
Senior	30.00 (0.00–60.00)	40.00 (0.00–65.00)	33.33	*p*-value = 0.14				
Bikini	30.00 (0.00–60.00)	45.00 (0.00–60.00)	50	*p*-value = 0.04*	*p*-value = 0.42 *η*_H_^2^−0.00 small	*p*-value = 0.16 *η*_H_^2^ 0.07 moderate		
Figure	30.00 (0.00–60.00)	15.00 (0.00–60.00)	−50	*p*-value = NA				
Wellness	40.00 (0.00–65.00)	40.00 (0.00–125.00)	0	*p*-value = 0.03*				
Women's Physique	15.00 (0.00–30.00)	30.00 (0.00–40.00)	100	*p*-value = NA				

Mean (minimal–maximum); statistic difference by posthoc.kruskal.nemenyi.test between: (men: a, classic physique; b, bodybuilding classic; c, master; d, men's physique; e, senior); (women: a, wellness; b, women's physique; c, bikini; d, figure).

* *p* < 0.05.

## Discussion

The purpose of this study was to quantify the weekly training volume performed by physique athletes, determine differences between men and women, compare competitive categories across different muscle groups, and evaluate these findings in relation to current strength training (ST) recommendations in the literature.

The results revealed notable variations in training volume between muscle groups across off-season and pré-contest phases.

Our findings revealed variable training frequencies and volumes across muscle groups, with some remaining relatively stable between off-season and pré-contest periods, while others showed notable adjustments. Among men, muscle groups such as the pectorals, latissimus dorsi, biceps brachii, triceps, hamstrings, and triceps surae were trained with similar frequency in both periods. However, deltoids, abdominals, and lower back showed variation.

The increase in aerobic exercise during the pré-contest in most categories with the decrease in frequency for some muscle groups pré-contest, may be a contributing factor to the reduction in total weekly volume and aligns with the pré-contest adjustment strategy to manage training stress according to other study (3).

Previous studies (8, 17, 19) have shown that physique athletes typically maintain high training volumes for large muscle groups (e.g., pectorals) year-round, with dietary and cardiovascular modifications being more prominent during the pré-contest phase. Men generally performed a higher volume of upper body sets (47.2 ± 14.6) compared to women (18.2 ± 7.4), whereas women showed greater lower body volume (23.8 ± 11.2 vs. 11.5 ± 7.0 sets). Our findings confirmed these trends among competitive athletes. For example, Classic Bodybuilding athletes performed 20 chest sets weekly in the off-season and 25 in the pré-contest.

Given the scarce literature available at this level of specificity, in women, muscle groups such as the triceps surae and gluteus were trained more frequently than the lower back and abdominals.

Hormonal differences, especially testosterone and estradiol, affect protein synthesis and response to training, but both sexes respond positively to the stimulus.Women present specific molecular and metabolic responses, such as greater utilization of fatty acids during exercise, but this does not limit hypertrophy (20–23).

Men tend to have greater absolute gains in muscle mass due to greater initial muscle mass and higher testosterone levels, but relative gains (percentages relative to starting point) are very similar between the sexes (22, 24–27).

The Wellness category requires larger buttocks than Bikini and Figure, and often also more voluminous than Women's Physique, although the latter requires greater definition and density. This may explain a higher frequency for the Wellness and Women's Physique categories.

The development of the glutes must present a volume equivalent to the natural anatomical standard, without fillers and proportional to the category's requirements, with evident muscle definition, perfect symmetry, smooth skin and no cellulite, the result of training focused on compound and isolated exercises for hypertrophy, combined with nutrition that favors muscle gain with fat control, all highlighted by a presentation and pose that highlight the anatomically natural curvature and shape of the glutes. In Bikini the glutes are toned and smooth with little volume and slight definition for a natural and elegant appearance, in Wellness there is greater volume and more marked curves with moderate definition for an athletic and curvy physique, while in Women's Physique the glutes are strongly muscled, dense and well defined, integrated into an athletic and muscular body with high definition.

Several factors, including training goals, individual preferences, and physiologicalneeds, may explain the observed variability in training frequency and volume.

However, to date, no specific recommendations exist for highly trained individuals, highlighting the significance of this study to our knowledge, this is one of the first studies to quantitatively describe the practices of physique athletes at this level of specificity, divided by categories, federations and genders.

### Results by category

The present findings revealed significant differences in weekly set volume between upper body, lower body, and core muscle groups across categories and training phases. Specific differences include:

• Men's Physique: Reduced training volume for the pectoral muscles during pré-contest.
• Classic Physique and Men's Physique: Reduced volume for the deltoids during pré-contest.
• Bodybuilding Classic vs. Men's Physique: Differences in pectoral and deltoid volume during off-season. Men's Physique trained more.
• Master Category: Increased training volume for the quadriceps during pré-contest.
• Bodybuilding Classic and Master Categories: Increased training volume for the abdominals during pré-contest.
• Classic Physique vs. Men's Physique: Differences in triceps surae volume during pré-contest. Classic Physique trained more.

These findings suggest a common adaptation strategy where training volume is reduced during the pré-contest period to prioritize recovery and maximize aesthetic outcomes for competition. Adjustments in training variables during the pre-contest phase align with previous literature, which highlights the importance of balancing volume and recovery to achieve hypertrophy (36). There is a gradual dose-response relationship, in which increases in resistance training volume produce greater gains in muscle hypertrophy (36). In this particular study, since we did not analyze the amount of weight lifted, muscle groups with more sets performed may be intended to increase the total work performed to achieve more hypertrophy or may be intended to compensate for a reduction in weight lifted without decreasing the total volume of the training load in the pre-contest period.

Prolonged or intense training without adequate recovery leads to increased perceived exertion, worsened mood, and slower cognitive reaction times, all of which can reduce training quality and performance. Central fatigue (affecting the brain and central nervous system) often appears before peripheral fatigue (muscle-based), indicating that mental and neural factors can limit performance even before muscles are fully exhausted (28–31).

High training loads can cause central fatigue, characterized by reduced motivation, slower information processing, and impaired voluntary muscle activation. This can persist for up to 72 h after intense sessions, especially with strength, jump, or sprint training, although the primary cause of fatigue is not always central nervous system dysfunction (29, 30, 32, 33).

Benito et al. (34) identified that training volume exceeding 16 sets persession could inversely affect hypertrophic gains, emphasizing the importance of moderating volume during high-intensity training phases. Consistent with prior research (17, 19), this study also observed higher training volumes for upper body muscles in men and lower body muscles in women. Specifically, competitive athletes in this study reported performing an average of 20 sets per week for the pectoral muscles during the off-season, increasing to 25 sets during the pré-contest phase in the *Bodybuilding Classic* category.

### Cardio training analysis

An increase in cardio training frequency up to 133% in frequency and 100% in duration per session as competition approached.

Cardio training frequency and session duration significantly increased during the pré-contest period, particularly in categories such as *Classic Physique* (12,50%), *Bodybuilding Classic* (33.33%), *Bikini* (50%), and *Wellness* (0% in the media but with a significant difference between specific athletes) (*p* < 0.05). This increase aligns with the need to reduce body fat and improve conditioning as competition approaches (37).

· *Bikini* category athletes performed cardio sessions at a significantly higherweekly frequency compared to the *Women's Physique* category. *Classic Physique* athletes exhibited a greater frequency of cardio sessions compared to *Senior* category athletes during pré-contest.

These findings reflect category-specific aesthetic goals, with *Men's Physique* competitors generally aiming for a leaner, less muscular physique, while *Women's Physique* athletes strive for more muscular and defined body compositions.

All Athletes need to reduce body fat before the contest, seeking maximum muscle definition, which is defined according to the specific needs of each category. Using aerobic activity can be interesting to increase energy expenditure without having to change training by muscle group even more.

Prior research supports the effectiveness of cardio training in reducing body fat and Improving overall body composition in physique athletes (35, 37).

The relationship between aerobic exercise and hypertrophy training can be either synergistic or antagonistic, depending on factors such as intensity, modality, volume and training schedule. In general, aerobic exercise does not significantly compromise muscle hypertrophy when well planned, but it can attenuate gains in specific situations. In this study, the data analyzed may reflect a need to increase caloric expenditure to enhance fat burning.

### Athletes' needs analisys

In the men's bodybuilding categories, the muscular standard varies according to the competitive focus, ranging from more classic physiques to extremely muscular ones.

In Men's Physique, the goal is to achieve an athletic and aesthetically pleasing body, with emphasis on chest, back, and abdominal development, featuring a V-shape appearance. The legs are not heavily judged since athletes wear board shorts, although calves may be used as a tiebreaker criterion.

In Classic Physique and Bodybuilding Classic, the objective is to resemble the bodybuilding style of the 1970s and 80s, showcasing solid muscle mass, symmetrical lines, and an “X” aesthetic—narrow waist, broad shoulders, and well-developed legs, while maintaining proportion and definition, but with a weight limit proportional to height. There is strong emphasis on muscle density, separation, and extreme conditioning. In age-based categories, such as Senior and Master, the standards are similar to the main divisions, though there is less emphasis on a tight waistline and some adjustment for the level of conditioning expected with age.

In competitive bodybuilding, the “senior” category typically includes athletes aged 24–35 years and “masters” (over 35 for women, over 40 for men) categories. Still, a well-developed, symmetrical, and defined physique is required.

In the female categories, the criteria evaluate not only muscle development but also femininity, proportion, and overall aesthetics.

Bikini Fitness is the category with the lowest muscle volume requirement, emphasizing natural curves, light definition, a slim waist, and toned glutes, without pronounced muscle separation. The look is elegant, with a more commercial appeal.

In Wellness Fitness, there is a clear focus on lower body development—well-defined glutes, quadriceps, and hamstrings, with less muscle volume in the upper body. The level of definition is more pronounced than in Bikini, but still without extreme vascularity or muscular hardness.

In Women's Physique, the muscular standard is higher: the goal is a physique with significant lean mass, sharp muscle definition, symmetry, and body control. Athletes display strength and muscle expression through mandatory poses, combining power with aesthetics. Physique athletes shape their training practices aiming to meet the specific standards of their respective categories as closely as possible in order to reach the podium.

Therefore, the findings reported in this study support the achievement of the requirements described above.

## Conclusion

This study highlights the individualized nature of training practices among physique athletes, with variations in training volume and frequency depending on competitive categories and sex. Notable reductions in training volume were observed during the pré-contest period for some muscle groups e.g.,: pectoral, deltoids, likely to enhance recovery and optimize aesthetics for competition.

This study did not analyze total training load based on lifted weight, but rather total weekly volume, representing a limitation. Future research should incorporate total workload analysis to better inform training strategies for physique athletes. For now, we recommend increased cardiovascular training during the pré-contest period and reduced total training volume for some muscular groups—an approach supported by current evidence.

Female athletes should prioritize lower body development, while male athletes should emphasize upper body musculature, aligned with category-specific demands.

Men's Physique athletes may benefit from reducing chest and deltoid training to avoid overdevelopment and emphasize definition. For Classic Physique athletes, reduced deltoid volume helps preserve proportionality, while abdominal and quadriceps training should be emphasized.

Classic Bodybuilding athletes tend to perform fewer chest sets than Men's Physique athletes during the off-season. In the Master category, increased quadriceps and abdominal volume may counteract age-related muscle loss. Overall, athletes should adjust training volume and frequency approaching competition to optimize recovery and physique. These adjustments must be individualized.

This study provides a novel contribution by systematically analyzing training volume in competitive physique athletes, offering a foundation for future research and evidence-based coaching.

We suggest new studies that can help in the development of more specific training practices for bodybuilding athletes with measurement of weight lifted, recovery interval between each series, cadence, caloric intake or plasma markers.

## Data Availability

The raw data supporting the conclusions of this article will be made available by the authors, without undue reservation.
